# Screening of older patients for atrial fibrillation in general practice: Current evidence and its implications for future practice

**DOI:** 10.1080/13814788.2017.1374366

**Published:** 2017-10-16

**Authors:** Matthew R. Fay, David A. Fitzmaurice, Ben Freedman

**Affiliations:** a Westcliffe Practice Group and Westcliffe Cardiology Service, Shipley, West Yorkshire, UK;; b Warwick Medical School, University of Warwick, Coventry, UK;; c Heart Research Institute, Charles Perkins Centre, The University of Sydney, Sydney, Australia

**Keywords:** Heart and circulation, general practice, diagnosis, public health in the community, healthcare technology

## Abstract

**Background:** Individuals with atrial fibrillation (AF) face a fivefold increased risk of ischaemic stroke compared with those without the condition. Recent studies suggest that individuals with asymptomatic AF also face an increased risk of ischaemic stroke, but their condition is often not recognized and diagnosed until an ischaemic stroke event has occurred. Identification of individuals with undiagnosed AF at increased risk for stroke is critical in promoting optimal intervention with anticoagulants.

**Objectives:** In this narrative review, we consider the benefits and limitations of various proposed screening strategies, whether single or multiple time-points, in addition to devices for implementation in the primary care setting.

**Outcomes:** Opportunistic screening via pulse palpation with subsequent referral for 12-lead electrocardiogram testing has been shown to cost-effectively identify individuals with asymptomatic AF. Some handheld devices suitable for use in primary care settings are now available and may facilitate screening of large cohorts of individuals considered to be at increased risk of AF, such as those aged ≥65 years or those diagnosed with or undergoing monitoring for hypertension.

**Conclusions:** It was determined that improved detection and diagnosis of AF, combined with appropriate anticoagulation strategies, will be crucial for improving stroke prevention and reducing its associated social and economic costs.

KEY MESSAGES

- Asymptomatic atrial fibrillation (AF) increases the risk of ischaemic stroke but is often only diagnosed after stroke occurs.
- Active, opportunistic screening in patients at risk of AF increases detection rates of asymptomatic AF.
- Screening for asymptomatic AF may reduce the risk of stroke through the appropriate use of anticoagulant therapy.

## Introduction

In 2010, it was estimated that more than 30 million people worldwide had a diagnosis of atrial fibrillation (AF) [1]. The prevalence of AF increases with advancing age, and the burden of AF is predicted to grow rapidly with the ageing global population [2,3]. AF is associated with substantial social and economic costs, in part because it is a major cause of ischaemic stroke. Depending on the presentation, duration and termination of AF episodes, AF is traditionally classified as follows: newly diagnosed, paroxysmal (self-terminating), persistent (lasts longer than seven days), long-standing persistent (continuous for more than one year before electing to undergo cardioversion), and permanent AF (accepted by the patient and physician) [4]. Although it is acknowledged that these categories are not mutually exclusive, it is useful to categorize patients based on their most frequent pattern of presentation [5].

A study of data from the Swedish Riks-Stroke register found that one-third of all patients with ischaemic stroke had previously known or newly diagnosed AF [6]. AF-related stroke is, on average, more severe than non-AF-related stroke and is associated with worse outcomes and increased mortality [7,8]. Each stroke has been estimated to cost the UK National Health Service (NHS) £15 306 over the first five years [9], and these figures may be even higher for AF-related strokes because they are on average more severe. To reduce the risk of stroke, clinical guidelines recommend the use of anticoagulation therapy in patients categorized as medium or high risk using the CHA_2_DS_2_-VASc scoring system [4,10–12].

Early and accurate diagnosis of AF is an essential step in gaining protective coverage from anticoagulation therapy, which may prevent an initial ischaemic stroke event. Most patients experience AF as a chronically progressive disease that eventually becomes permanent [4]. A 2015 study of patients through the UK Clinical Practice Research Datalink found that 39% of patients with incident AF were first diagnosed in general practice, with the remainder diagnosed in hospital [13]. Unfortunately, in many patients diagnosis occurs after a stroke event has taken place. A meta-analysis of 32 studies found an overall AF detection rate of 11.5% in patients with no known AF after an ischaemic stroke or transient ischaemic attack [14]. Therefore, a considerable number of individuals may unknowingly be at increased risk of a disabling and potentially life-threatening stroke. A major diagnostic challenge relates to those with paroxysmal or asymptomatic (silent) AF, particularly given the results of the ASSERT and Copenhagen Holter studies, which clearly indicate that even short episodes of ‘silent’ AF are associated with an increased risk of stroke [15,16]. The recent Olmsted country study stated that over 50% of patients with AF in the community present with atypical or no symptoms and these forms of AF may be more hazardous with regards to clinical outcomes [17]. However, at present, the minimum burden of paroxysmal AF needed to justify anticoagulation therapy remains unknown.

To ensure that all eligible patients with AF receive appropriate interventions to mitigate their risk of stroke, there is a recognized need to improve AF detection rates before the first complications arise and to provide adequate anticoagulation [4]. The development of effective screening strategies for AF requires appropriate target populations to be determined (population level, those over a defined age or, for example, and those attending for blood pressure screening). The timing must also be considered (i.e. a single time-point or regular screening), to provide the best chance of identifying patients with paroxysmal AF in the most convenient and cost-effective manner for primary care. In this review, AF detected via single time-point screening or a patient-activated recorder will be referred to as screen-detected AF. Appropriate diagnosis and subsequent anticoagulation therapy in patients with previously undetected AF can significantly reduce the incidence of an initial ischaemic stroke event and its associated healthcare and human costs.

In this context, this review examines the evidence for active AF screening and the benefits and limitations of various proposed screening strategies and devices in primary care. Current barriers to widespread implementation of these strategies are also explored.

## Atrial fibrillation screening studies: current clinical and economic findings

### Single time-point screening

Many studies have reported on the benefits of single time-point screening of older patients for unidentified AF using an electrocardiogram (ECG) or pulse palpation [18]. In 2007, the large-scale screening for AF in the elderly (SAFE) study showed that active screening for AF (opportunistic or systematic) among patients aged ≥65 years (mean ± standard deviation [SD] age: 75.3 ± 7.2 years) was indeed more effective in detecting AF than routine care [19]. Across 50 primary care centres in England, almost 15 000 individuals were recruited and were either actively screened for AF (pulse palpitation followed by 12-lead ECG if the pulse was irregular) or continued with routine care. The AF detection rate was 1.63%/year in the systematically screened group versus 1.04%/year in the control group (*p* <0.05). The SAFE study also showed that single time-point opportunistic strategies (irregular pulse on pulse check, with ECG to confirm) and systematic screening (invited for ECG) yielded very similar results to each other regarding AF detection rates [19]. An economic evaluation within the trial showed the lowest incremental cost was for opportunistic screening (£337 for each additional AF case identified) [20].

A systematic review of 30 individual studies (including SAFE) of single time-point screening (ECG or pulse palpation) in >122 000 patients reported an AF prevalence of 2.3%, rising to 4.4% in patients aged ≥65 years [18]. The overall incidence of screen-detected AF was 1.0%, increasing to 1.4% among patients aged ≥65 years regardless of whether studies were performed in the clinic or the community. Mean stroke risk in patients with screen-detected AF was shown to be moderate to high (mean CHA_2_DS_2_-VASc score of 3.3, increasing to 3.8 in patients aged ≥65 years; based on data from two studies). Of the three studies that reported eligibility for oral anticoagulation, 67% of patients with screen-detected AF were indicated for oral anticoagulation [18].

## Handheld devices

Several studies have used handheld devices to facilitate opportunistic screening for AF (Boxes 1–3, Table 1). In 2014, the SEARCH-AF study investigated community AF screening in Australia between 2012 and 2013 using the AliveCor™ handheld ECG device (AliveCor Inc., San Francisco, USA) in 1000 unselected subjects aged ≥65 years who attended their local pharmacies [21]. Screen-detected AF was reported in 1.5% of subjects; all those identified had a CHA_2_DS_2_-VASc score ≥2, indicating that oral anticoagulation would be beneficial for stroke prevention. The incremental cost-effectiveness ratio for extending the iPhone-based ECG to the community was calculated as AU$5988 (€3142 using 2013 estimates) per quality-adjusted life-year (QALY) gained, and AU$30 481 (€15 993) per stroke avoided, which would be considered very cost-effective. Another recent study used a MyDiagnostick™ handheld bar device (Applied Biomedical Systems BV, Maastricht, The Netherlands) to screen for AF in patients visiting their general practice for influenza vaccination [22]. As a result of this screening, AF was detected in 1.1% of patients, 78.4% of whom had a CHA_2_DS_2_-VASc score ≥2. Furthermore, a cost-effectiveness analysis has been performed, showing that use of the MyDiagnostick™ device to screen patients aged ≥65 that attend seasonal influenza vaccination had a 99.8% chance of being cost-effective [23].

**Table 1.** A selection of devices suitable for opportunistic screening for AF in the primary care setting.

Device and manufacturer	Description	Validation and diagnostic accuracy data	Recommended for use in the primary care setting?	Cost-effectiveness data available?	Convenience of use
WatchBP *Microlife*	BP cuff Automated BP monitor with inbuilt AF detection system.	– Six trials (*n* = 2332) using BP cuffs with the AF detection algorithm by Microlife [42] resulted in sensitivity and specificity values of 90–100%. – Unselected primary care population (= 1000, mean age 79.7 years) [24]: sensitivity: 94.9%, specificity: 89.7%.	Yes: NICE recommendation [43].	Yes: small cost-saving compared with pulse palpation [43].	Can be used at home; requires correct positioning of the cuff; patient must return device for data download.
MyDiagnostick *Applied Biomedical Systems*	Handheld ‘bar’ device one-lead ECG device	– Primary care population of patients with known paroxysmal or chronic AF (*n* = 161) and a convenience sample of individuals without AF (*n* = 30) [45]: sensitivity: 94%, specificity: 93% – Primary care influenza vaccination clinics, *n* = 676 patients attending a single clinic: diagnostic accuracy of 100% for patients with known AF and detection of AF in a further 11 patients not previously diagnosed [44]. – Across 10 Dutch primary care practices: 37 (1.1% of all those screened) new cases of AF detected [22].	No	Yes: lifetime costs and effects of a single screening session for AF detection in a community setting [23].	Can be used at home; patient must return device for data download.
Zenicor-ECG *Zenicor Medical Systems*	Handheld unit one-lead ECG device with two thumb sensors	– Evaluated in 22 patients diagnosed with symptomatic, paroxysmal AF, with patients asked to make two recordings per day for 30 days and additional recordings if arrhythmia symptoms arose [46]. Patients also underwent 24-h ambulatory monitoring. – Diagnosis of AF events: Intermittent monitoring: 18 patients (82%); 24-h monitoring: seven patients (32%; *p* = 0.001).	No	Cost-effectiveness of two weeks of intermittent screening for asymptomatic AF in 75/76-year-old individuals [26].	Recordings can be collected remotely via a server to facilitate clinical review.
AliveCor *AliveCor Inc.*	Sensors attached to smartphone or smartwatch.	– Among a validation set of 204 patients that included 48 with confirmed AF [47]: sensitivity: 98%, specificity: 97%, diagnostic accuracy: 97%. – Evaluated in 381 individuals including healthy young adults (*n* = 128), elite athletes (*n* = 123) and patients attending a cardiology clinic (*n* = 130) [48]: sensitivity in ambulatory cardiology patients: 94.4%, specificity: 100% in healthy young adults, 99.2% in elite athletes and 99.1% in ambulatory cardiology patients.	No	Cost-effectiveness of community screening increased with improved anticoagulant therapy adherence [21].	Recordings can be collected remotely via a server to facilitate clinical review.
Cardiio Rhythm *Cardiio, Inc.*	PPG smartphone app	– Evaluated in 1013 ambulatory patients from a general outpatient clinic aged ≥65 with hypertension and/or diabetes [37]: sensitivity: 92.9%, specificity: 97.7%.	No	No data available.	Can be used at home (positive results require verification by ECG).

BP: blood pressure.

**Box 1.** Automated blood pressure monitors for AF screening.

Several blood pressure monitors have been adapted using an embedded algorithm that detects pulse irregularities indicative of AF with a sensitivity and specificity between 90% and 100% using ECG diagnosis as a reference [42]. By contrast, sensitivity and specificity for pulse palpitation have been measured at 87.2% and 81.3%, respectively [20]. On the basis of these studies, NICE recommended the WatchBP^TM^ device (Microlife AF, Widnau, Switzerland; Table 1) for opportunistic screening of AF among individuals with suspected hypertension and those undergoing screening or monitoring for hypertension in the primary care setting [43]. The NICE evaluation identified a small estimated cost saving of £2.98 per person screened in those aged 65–74 years rising to £4.26 for those aged ≥75 years, compared with pulse palpation and subsequent referral for ECG, based on the costs of each modality and anticipated cost savings associated with stroke prevention [43]. In a separate study of 1000 elderly patients (mean age 79.7 years; range: 75.1–99.8 years) recruited from six general practices in the UK, the WatchBP device outperformed two single-lead ECG devices (Omron HCG-801 and Merlin ECG event recorder), achieving a sensitivity of 94.9% and a specificity of 89.7% for the detection of AF (95% confidence interval 87.5–91.6%) [24].

**Box 2.** Single-lead ECG devices for AF screening.

Handheld, single-lead ECG devices are an attractive option for AF screening because the resulting ECG can be analysed by a physician in the case of a positive result, eliminating the need for a follow-up ECG [44]. Such devices include: the MyDiagnostick (Applied Biomedical Systems BV, Maastricht, The Netherlands), a ‘bar’ device that generates a one-minute ECG when held by the user; the Zenicor-ECG (Zenicor Medical Systems AB, Stockholm, Sweden), a device that records a 30-s ECG when thumbs are placed on the sensors; and the AliveCor (AliveCor, Inc., San Francisco, USA), which consists of two sensors that attach to the back of a smartphone and an App that captures a 30-s ECG when the user’s fingers are placed on the electrodes. These devices and the results of associated validation studies, including sensitivity and specificity, are described in further detail in Table 1.

**Box 3.** Pulse photoplethysmographic (PPG) devices for AF screening.

Another approach to AF monitoring by smartphone is an App that uses the light source and camera of the phone to obtain a PPG recording of the pulse wave, then applies an algorithm to analyse its regularity. A recent study evaluated the ‘Cardiio Rhythm’ App (in development) (Table 1) and AliveCor device (Box 2) in ambulatory patients aged ≥65 years with hypertension and/or diabetes from a general outpatient clinic [37,38]. The results indicated a sensitivity of 92.9% and specificity of 97.7% for the app, compared with 71.4% and 99.4%, respectively, for the AliveCor device [38].

Together, these studies show that single time-point screening for AF can improve detection rates compared with routine diagnosis and that most patients with newly detected AF are at high enough stroke risk to require anticoagulant treatment.

## Multiple time-point screening

A potential issue with any single time-point screening strategy is the possibility of ‘missing’ an AF signal in patients with paroxysmal AF, who may not be experiencing an AF episode at the time of screening. Furthermore, although pulse palpation has a high degree of sensitivity for AF (87–97%), there is an element of subjectivity that may lead to false positives; additionally, its specificity is lower (70–81%) [24].

To address these potential limitations, the Swedish STROKESTOP study was the first multicentre, prospective study to employ a systematic screening programme using twice-daily ECGs obtained over a two-week period with the Zenicor-ECG™ handheld ECG recorder (Zenicor Medical Systems AB, Stockholm, Sweden; Box 2) to detect AF in patients aged 75 or 76 years [25]. The study found that 3% of participants who underwent screening had AF detected and a further 2.1% of participants had known AF but were not taking an anticoagulant: about half of these were subsequently commenced on an anticoagulant after referral to a clinic. Importantly, 93% of patients with newly diagnosed AF accepted oral anticoagulant therapy. An analytic simulation model based on the STROKESTOP population reported that intermittent screening of 75 and 76-year-olds for AF was cost-effective, incurring a cost of €4313 per QALY when a lifelong perspective was used [26]. The model showed that screening 1000 individuals could result in eight fewer strokes, 11 additional life-years, and 12 additional QALYs at an incremental cost of €6583 per stroke avoided.

## National screening programmes: what are the current barriers?

### Guideline recommendations

In 2012, both the European Society of Cardiology (ESC) and the Royal College of Physicians of Edinburgh recommended that opportunistic screening by pulse palpation, followed by confirmatory ECG, be considered for all patients aged ≥65 years [27,28]. In 2015, the European Primary Care Cardiovascular Society (EPCCS) also recommended opportunistic screening in patients aged ≥65 years by pulse palpation and confirmatory ECG, or by using modified sphygmomanometers or single-lead ECG devices if subject to independent validation with a 12-lead ECG [12]. Most recently, the 2016 update to the ESC guidelines for AF management reiterated the previous recommendation for opportunistic screening by pulse palpation or ECG in patients aged >65 years and suggested systematic ECG screening be considered in patients aged >75 years or at high stroke risk (Class IIb recommendation, level B evidence) [29]. The new ESC guidelines also recommend extended screening in patients after a transient ischaemic attack or ischaemic stroke, which should include a short-term ECG followed by at least 72 hours’ continuous ECG monitoring (Class I, level B). For stroke patients, further monitoring using long-term, non-invasive ECG monitors or implanted loop recorders to pick up silent AF episodes may be considered (Class IIa, level B). Furthermore, the guidelines recommended interrogating pacemakers and implantable cardioverter defibrillators on a regular basis to detect atrial high-rate episodes, which should trigger further investigation by ECG to document AF (Class I, level B) [29].

## Regulatory barriers

The UK National Institute of Health and Care Excellence (NICE) [30] and European ESC clinical guidelines do not differentiate between symptomatic and asymptomatic AF in terms of treatment [4]. However, in 2014 the National Screening Committee (NSC), who advise government ministers and the UK National Health Service (NHS) on all aspects of screening, did not recommend routine screening of the population aged >65 years, because ‘it is uncertain that screening will do more good than harm to people identified during screening for AF’. This was based on the observations that: (1) the treatment and care of people with AF was non-optimal; (2) that better evidence was needed on whether AF detected at screening carried the same long-term risk of stroke as AF found in the context of other conditions; and (3) that tests for AF needed to be improved and standardized [31].

This first observation is of considerable significance because, despite robust evidence-based recommendations, recent registry data suggests that although there is a trend towards increased prescribing of oral anticoagulants up to 10% of patients with known AF do not receive appropriate anticoagulant therapy [32]. Any potential benefits of screening for AF will be dependent on making sure that eligible patients receive anticoagulant therapy for reducing the risk of stroke, with analyses such as SEARCH-AF showing that cost-effectiveness is related to subsequent adherence to guideline-recommended anticoagulant prescription [21].

The second observation, however, can perhaps be addressed based on the current evidence base. In the Olmsted County study, 34% of patients with confirmed AF were asymptomatic at the time of diagnosis but were almost three-times more likely to experience a cerebrovascular event than patients with typical AF [17]. Another study that followed a cohort of 5555 patients with asymptomatic, incidentally detected AF identified a stroke incidence rate of 19.4/1000 patient-years, compared with 8.4/1000 patient-years in control individuals [33]. Asymptomatic AF that may be detected through a screening programme is, therefore, clearly not ‘benign’ and warrants detection and treatment before a stroke occurs [34]. Moreover, a secondary analysis of the SAFE study population reported that the stroke risk profiles of patients detected via opportunistic (pulse and ECG) and systematic (postal invitation for ECG) screening were very similar [35]. They reported that 83% of patients identified through opportunistic screening and 78% of those identified through systematic screening had stroke risk profiles that favoured anticoagulation. The STROKESTOP authors also pointed out that AF aligns with the 10 principles set out by the World Health Organization, which provide rationalization for mass screening (Table 2) [25,36].

**Table 2.** World Health Organization: principles of early disease detection [36].

Principles
The condition sought should be an important health problem.
There should be an accepted treatment for patients with recognized disease.
Facilities for diagnosis and treatment should be available.
There should be a recognizable latent or early symptomatic stage.
There should be a suitable test or examination.
The test should be acceptable to the population.
The natural history of the condition, including development from latent to declared disease, should be adequately understood.
There should be an agreed policy on whom to treat as patients.
The cost of case-finding (including diagnosis and treatment of patients diagnosed) should be economically balanced in relation to possible expenditure on medical care as a whole.
Case-finding should be a continuing process and not a ‘once and for all’ project.

## Device limitations

Whether for use at home or in a primary care setting, the appropriate choice of device will be crucial to maximize the cost-effectiveness of a screening programme. Interestingly, a recent study comparing the Cardiio Rhythm™ App (Cardiio Inc., Cambridge, USA; Box 3) to the AliveCor device (Box 2) measured a sensitivity for the latter device of 71.4% and a specificity of >99% [37]. Although the specificity was high, the sensitivity was substantially lower than that measured for AliveCor in its validation studies (Table 1). This difference reflects an intentional algorithm change to favour greater specificity over sensitivity [38]. This trade-off between sensitivity and specificity for handheld monitoring devices is an important factor in considering which device is most appropriate for home use versus a community care setting [38].

## Operating staff

Successful screening using any of the devices described will require adequate training for healthcare staff if they are to be used in an office setting and for patients if they are to be used in a home setting for multiple time-point screening. A pilot study, GP-SEARCH, has provided initial insights into how confident general practitioners (GPs), nurses and receptionists were in the use of iPhone ECG (iECG) devices for AF screening [39]. The study was conducted in three primary care practices in Australia, with receptionists and practice nurses screening patients aged ≥65 years using an iECG device (AliveCor; Box 2), and a GP reviewing the data. Although nurses were confident in the use of the iECG device, receptionists expressed a reluctance to use the device and were not confident to offer screening or explain the need for the screening to patients. Patients themselves were accepting of the iECG device. A follow-up study investigating AF screening by nurses using an iECG device in a practice setting again highlighted practice-specific barriers that were mainly related to staff time and funding [40].

Another recent study surveyed the views of healthcare practitioners in inner-city GP surgeries in Nottingham, UK, to ascertain their readiness for future screening in AF [41]. The majority of practices surveyed were able to perform and interpret ECGs in-house, but they highlighted the need for additional training. Other barriers to screening implementation included the time to perform the screening, workload, staffing levels within the practice and available funding to conduct screening activities.

## Conclusions

As the global population continues to age, the diagnosis and management of conditions such as AF and AF-related stroke will account for an increasing proportion of the healthcare budget. The total direct human cost of AF-related stroke will also increase as proportionally more individuals experience these debilitating and life-threatening events. Large-scale screening to identify individuals with AF as a prelude to stroke risk assessment and use of appropriate interventions to mitigate the risk will be critical to address these future challenges. Current screening strategies aim to increase coverage of patient cohorts, including patients aged ≥65 or with ongoing monitoring of hypertension, using a variety of convenient handheld devices, which have been summarized in this review. However, widespread use of such strategies is limited by a lack of appropriate funding, high staff workloads, staffing levels and lack of confidence with devices.

Improved AF detection and diagnosis, whether by pulse palpation or convenient handheld devices, will amount to little if adequate treatment strategies are not implemented. Risk assessment and the appropriate use of anticoagulants, including non-vitamin K antagonist oral anticoagulants (NOACs), will be essential to translate the potential benefits of increased detection into improved stroke prevention, with the associated societal cost benefits and a reduction in the direct human costs of AF-related stroke.
